# Differential willingness for genetic testing to target treatment in older Danish citizens

**DOI:** 10.1093/eurpub/ckac131.554

**Published:** 2022-10-25

**Authors:** A Leppin, JB Nielsen

**Affiliations:** 1 Unit Health Promotion, Department of Public Health, University of Southern Denmark, Esbjerg, Denmark; 2 Unit General Practice, Department of Public Health, University of Southern Denmark, Odense, Denmark

## Abstract

**Background:**

Studies from different countries have shown that population majorities are willing to accept genetic tests for treatment personalisation and that considerable proportions are ready to donate their data to research. However, it has also been shown that concerns, for example about data use and confidentiality or treatment rationing, are common. To enable a more targeted communication process with the public about personalized medicine, more knowledge is needed on views in different sub-populations. In the present study, a hypothetical scenario was used to investigate differential readiness to accept a genetic test for treatment targeting and to permit use of personal data for research.

**Methods:**

A cross-sectional survey was conducted with 50-80-year-old Danish citizens (n = 6807) who were sampled to represent the Danish population in that age segment. Socio-demographic data were added from a national registry. Data were analyzed by multivariable logistic regression analysis.

**Results:**

Preliminary results showed that a majority was willing to be tested (78.3%). Readiness was lower in women [OR = .67; CI = .59-.77] and those 70-80 [OR = .72; CI = 61-.86], while it was higher in those with better income [OR = 1.29; CI = 1.09-1.52]. Further, those less satisfied with their health, the obese and those with a perceived genetic vulnerability were more willing to be tested. Over 90% of those ready to be tested were also willing to permit use of their data for research. Rates were higher in men, older segments, those with higher income/education as well as those with current pain experience and those aware of a personal genetic vulnerability.

**Conclusions:**

Findings indicate group differences in acceptance of a genetic test for personalisation of medicine and data use for research. Further research should investigate group-specific benefit perceptions versus concerns in population subgroups to inform implementation and enable targeted communication strategies.

**Key messages:**

• Acceptance of genetic testing for personalisation of treatment as well as willingness to contribute data to research may differ between population subgroups.

• Women and those with lower income are less willing to accept genetic testing for treatment personalisation and accept research use of data while health vulnerabilities increase acceptance.

